# Risk stratification of acute symptomatic anterior circulation intracranial atherosclerotic stenosis using an ABCD^3^-I-informed clinical-imaging score: an exploratory study

**DOI:** 10.3389/fneur.2026.1897338

**Published:** 2026-09-11

**Authors:** Zhuli Peng, Xibu Zhou, Zini Xiong, Haolin Liu, Xiaolong Chen, Xiaoxin Bai

**Affiliations:** Department of Cerebrovascular Diseases, Guangdong Provincial Hospital of Chinese Medicine (The Second Clinical College of Guangzhou University of Chinese Medicine), Guangzhou, China

**Keywords:** intracranial atherosclerotic stenosis, ABCD^3^-I score, risk stratification, early neurological deterioration, 90‑day outcomes, watershed infarction

## Abstract

**Background:**

Acute symptomatic anterior circulation intracranial atherosclerotic stenosis (ICAS; ≥70%) carries substantial risks of early neurological deterioration (END) and disability. We developed an ABCD^3^-I-informed clinical-imaging score incorporating pre-baseline progressive stroke, responsible-vessel stenosis, and diffusion-weighted imaging (DWI) infarct pattern for acute risk screening.

**Methods:**

We retrospectively enrolled 133 consecutive patients (103 men, 30 women; mean age: 64.62 ± 10.56 years) with acute symptomatic anterior circulation ICAS (≥70% stenosis; onset ≤7 days) at a single center (January 2024–June 2025). The score (0–13) categorized patients as high risk (≥8; *n* = 102) or non-high risk (*n* = 31). The primary outcome was the ordinal 90-day modified Rankin Scale (mRS) score; the secondary outcomes were mRS scores ≥3 and END. Adjusted regression and receiver operating characteristic (ROC) analyses were performed, and discrimination was compared with the original ABCD^3^-I.

**Results:**

The high-risk group had higher admission National Institutes of Health Stroke Scale (NIHSS) scores (median 2.0 vs. 0.0; *p* < 0.001). The ABCD^3^-I-informed clinical-imaging score discriminated END (area under the curve [AUC] 0.833, 95% CI 0.760–0.906; 50.0% vs. 19.4%, *p* = 0.005). The unadjusted 90-day mRS score distribution was worse in the high-risk group (z = −2.405, *p* = 0.016), but the ABCD^3^-I-informed clinical-imaging score was not significant after adjustment for admission NIHSS in the proportional-odds model (adjusted common OR 1.05 per point, 95% CI 0.88–1.26; *p* = 0.573). The dichotomized disability rate (mRS ≥ 3: 18.6% vs. 9.7%) did not differ significantly (*p* = 0.285; adjusted OR 1.11, 95% CI 0.86–1.44). For 90-day disability, the AUC was 0.662 (95% CI 0.540–0.783; *p* = 0.017). At the prespecified ≥8-point threshold, 31 patients (23.3%) were classified as non-high-risk; the negative predictive value was 90.3% (95% CI 75.1–96.7%). Discrimination did not differ significantly from the original ABCD^3^-I score (ΔAUC 0.068, 95% CI − 0.063 to 0.196; DeLong *p* = 0.312).

**Conclusion:**

The ABCD^3^-I-informed score may identify a non-high-risk subgroup and show strong END discrimination, but its association with a 90-day outcome was modest and NIHSS-dependent. It should complement, not replace, baseline neurological assessment and should not guide treatment selection; prospective multicenter validation is required.

## 1 Introduction

Symptomatic intracranial atherosclerotic stenosis (sICAS) is a leading cause of recurrent stroke and disability worldwide, with a disproportionately high prevalence in Asian populations compared with Western cohorts. Among these patients, those presenting with acute, severe (≥70%) stenosis of the anterior circulation are at particularly high risk of stroke progression and early recurrence. Despite guideline-recommended therapy, these patients remain at substantial risk of neurological deterioration and recurrent ischemia, highlighting the need for early risk stratification (1, 2).

Currently available risk assessment tools, including the ABCD^2^ and ABCD^3^-I scores, were predominantly developed and validated in transient ischemic attack (TIA) populations. These instruments do not adequately account for the degree of intracranial arterial stenosis or specific infarction patterns, both of which are critical determinants of prognosis in patients with severe ICAS (3). Although the ABCD^3^-I score has demonstrated good discriminative ability for predicting early stroke risk after TIA, its prognostic value in the specific setting of acute symptomatic severe intracranial stenosis has not been systematically evaluated. Moreover, the ABCD^3^-I score does not incorporate hemodynamic imaging biomarkers such as watershed infarction, which indicates hypoperfusion mechanisms and is closely associated with the risk of stroke recurrence.

In a previous 152-patient cohort, acute watershed infarction, severe responsible-vessel stenosis, and the ABCD^3^-I score independently predicted END, and a combined nomogram achieved an AUC of 0.85 (4). These findings supported adapting the ABCD^3^-I framework to incorporate intracranial stenosis severity and DWI-defined infarct pattern.

Building upon these preliminary findings, this study evaluated a prespecified ABCD^3^-I-informed clinical-imaging score that broadened the original dual TIA item to include pre-baseline progressive stroke and re-specified the two imaging items to reflect responsible-vessel stenosis severity and DWI-defined infarct pattern. The score was assessed in an independent cohort, focusing on 90‑day functional outcomes, exploratory findings within the intensive medical therapy (IMT) and endovascular treatment (EVT) subgroups, and incremental discrimination over the original ABCD^3^‑I score.

## 2 Materials and methods

### 2.1 Study design and patient population

This single-center, retrospective, observational cohort study included consecutive patients admitted to the Cerebrovascular Disease Unit of Guangdong Provincial Hospital of Chinese Medicine between January 2024 and June 2025 with acute symptomatic severe intracranial atherosclerotic stenosis of the anterior circulation. Of the 150 initially screened patients, 17 were excluded (pre-stroke modified Rankin Scale [mRS] score >2, *n* = 3; age >80 years, *n* = 2; non-atherosclerotic etiology, *n* = 4; terminal systemic illness, *n* = 2; loss to follow-up or missing key data, *n* = 6), yielding a final analytic cohort of 133 patients. This cohort was entirely independent of the previously reported 152-patient cohort, with no patient overlap.

The study protocol was approved by the Medical Ethics Committee of Guangdong Provincial Hospital of Chinese Medicine (Approval No. YE2025-029-01). Given the retrospective design, the requirement for written informed consent was waived by the same committee.

### 2.2 Diagnostic criteria and eligibility

#### 2.2.1 Diagnostic criteria

Transient ischemic attack (TIA) was defined as a transient episode of focal neurological dysfunction lasting less than 24 h without evidence of acute ischemic lesions on neuroimaging (5). Acute ischemic stroke was defined as an episode of neurological dysfunction resulting from focal infarction of the cerebral, spinal, or retinal tissue. Central nervous system (CNS) infarction was ascertained on the basis of neuropathological examination, neuroimaging findings, and/or clinical evidence of permanent tissue injury, in accordance with the American Heart Association/American Stroke Association (AHA/ASA) consensus statement (6).

The degree of intracranial arterial stenosis was quantified using the Warfarin–Aspirin Symptomatic Intracranial Disease (WASID) method (7): stenosis rate = (1 − minimal luminal diameter at the stenotic site/diameter of the normal reference segment) × 100%. The proximal normal arterial segment was used as the reference; if the proximal segment was diseased, the distal normal segment was used instead. Severe stenosis was defined as 70–99% luminal narrowing.

Acute infarct patterns were determined using DWI and fluid-attenuated inversion recovery (FLAIR), with vascular status assessed using transcranial Doppler (TCD), magnetic resonance angiography (MRA), computed tomography angiography (CTA), or digital subtraction angiography (DSA) to confirm that the lesion was attributable to the stenotic vessel. The DWI lesion pattern was classified into three mutually exclusive categories: (1) watershed infarction—lesions involving the cortical (external) and/or internal (subcortical) border zones of the anterior circulation, with or without concomitant cortical or perforator infarcts; (2) non-watershed infarction—single or multiple cortical or subcortical lesions confined to a single arterial territory without border-zone involvement; and (3) no acute DWI lesion (8–10). All DWI images were independently reviewed by two senior neurologists blinded to treatment allocation and clinical outcomes. Interrater reliability was excellent (Cohen’s κ = 0.929; overall agreement rate, 95.5% [127/133]), with discrepancies resolved by consensus.

#### 2.2.2 Inclusion criteria

Patients were eligible for inclusion if they met all the following criteria:

1. Age 18–80 years
2. Symptom onset to hospital admission ≤7 days
3. Anterior circulation intracranial stenosis ≥70% confirmed by MRA, CT angiography (CTA), or digital subtraction angiography (DSA), with TIA or acute ischemic stroke attributable to the territory of the stenotic vessel
4. Atherosclerotic etiology confirmed according to the modified Trial of Org 10172 in Acute Stroke Treatment (TOAST) classification
5. Pre-morbid mRS scores ≤2
6. No pre-existing impairment that would preclude reliable neurological or functional assessment
7. Receipt of guideline-directed medical therapy, including dual antiplatelet therapy, high-intensity statin, and individualized blood pressure and glucose management according to the current American Heart Association/American Stroke Association (AHA/ASA) guidelines

#### 2.2.3 Exclusion criteria

Patients were excluded if any of the following conditions were present:

1. Neuroimaging of insufficient quality for analysis or missing key clinical data
2. Hemorrhagic stroke
3. Cardioembolic source, small-vessel occlusive disease, other determined etiology, or undetermined etiology according to the modified TOAST classification
4. Requirement for long-term anticoagulation
5. Terminal systemic illness or expected survival <3 months
6. Asymptomatic intracranial stenosis or inability to complete follow-up

#### 2.2.4 Criteria for endovascular treatment

Urgent cerebral angiography within 72 h was indicated if any of the following were present:

1. Progressive or fluctuating neurological symptoms
2. A hemodynamically stable presentation with CTP evidence of hypoperfusion in the responsible-vessel territory, characterized by prolonged mean transit time/time-to-maximum and reduced cerebral blood flow compared to the contralateral homologous region
3. CTP evidence of hypoperfusion accompanied by clinical deterioration during close observation, defined as an increase in the NIHSS score of ≥2 points within 2–4 h and attributed to hypoperfusion based on clinical and imaging evaluation

Among patients who underwent angiography, EVT was considered when preprocedural DSA demonstrated impaired downstream perfusion, including incomplete distal filling, delayed contrast clearance, a pronounced shift of watershed territories, or reduced inflow velocity.

### 2.3 ABCD^3^-I-informed clinical-imaging score and patient grouping

Our previous studies identified three independent predictors of early neurological deterioration (END): acute watershed infarction, severe stenosis of the responsible vessel, and the ABCD^3^-I score (4). The original ABCD^3^-I score was calculated strictly according to the published instrument, with its imaging domain comprising ipsilateral carotid stenosis ≥50% on cervical artery imaging (2 points) and DWI hyperintensity (2 points). From that instrument, we prespecified an ABCD^3^-I-informed clinical-imaging score (hereafter “the score”; range, 0–13) with one clinical-domain adaptation and two imaging-domain adaptations. The original dual TIA item was broadened to include progressive stroke occurring before baseline assessment. In the imaging domain, carotid stenosis was replaced by anterior circulation intracranial stenosis (≥70%, 2 points; 50–69%, 1 point), and the binary DWI item was replaced by a pattern-graded item (watershed, 2 points; non-watershed, 1 point). All other clinical items and the total score range (0–13) were retained (Table 1). The stenosis item is graded so that the score can be applied across the full moderate-to-severe range, although the present cohort included only patients with severe stenosis (≥70%).

**Table 1:** Components and scoring of the ABCD^3^-I-informed clinical-imaging score.

Component	Criteria	Points	Score
A – Age	≥60 years	1	
B – Blood pressure	SBP ≥ 140 mmHg and/or DBP ≥ 90 mmHg	1	
C – Clinical features	Unilateral weakness	2	
	Speech disturbance without motor weakness	1	
D – Duration of symptoms	≥60 min	2	
	10–59 min	1	
D – Diabetes mellitus	Present	1	
D – Dual TIA / progressive stroke	≥1 additional TIA within 7 days of the index event or progressive neurological worsening (NIHSS score increase ≥2 points from onset up to the baseline assessment)	2	
I – Imaging	Anterior circulation ICAS ≥70%	2	
	Anterior circulation ICAS 50–69%	1	
	DWI hyperintensity with watershed infarction	2	
	DWI hyperintensity without watershed infarction	1	
Total score		13	

ICAS, intracranial atherosclerotic stenosis; TIA, transient ischemic attack; DWI, diffusion-weighted imaging; NIHSS, National Institutes of Health Stroke Scale; SBP, systolic blood pressure; DBP, diastolic blood pressure.

Regarding weighting, the original ABCD^3^-I clinical-item weights were retained, with pre-baseline progressive stroke included within the dual TIA item at 2 points. The two imaging features were selected based on their associations with END in the prior cohort (watershed infarction, odds ratio [OR] 8.21; severe responsible-vessel stenosis, OR 3.91). In the re-specified imaging domain, ≥70% stenosis and watershed infarction were assigned 2 points, whereas 50–69% stenosis and non-watershed DWI lesions were assigned 1 point. These pragmatic integer weights were anchored to the ABCD^3^-I framework rather than derived from regression coefficients or statistically optimized.

The score was calculated at the baseline (admission) assessment using only information available at that time; the progressive-worsening item captured an NIHSS score increase of ≥2 points between symptom onset and that assessment, whereas any subsequent deterioration was evaluated as the END outcome rather than scored. Risk was categorized as low (0–3), intermediate (4–7), or high (8–13 points), and patients were dichotomized for analysis into high-risk (≥8; *n* = 102) and non-high-risk (<8; *n* = 31) groups.

### 2.4 Treatment assignment

The choice between IMT and EVT was made by the treating clinician based on each patient’s clinical status, hemodynamic evaluation, and individual preference; treatment allocation was not randomized. Therefore, comparisons between the IMT (*n* = 74) and EVT (*n* = 59) subgroups are descriptive in nature. EVT consisted of DSA-guided balloon angioplasty with or without stent implantation and was performed under local or general anesthesia. Successful revascularization was defined as residual stenosis ≤50% and an expanded Treatment in Cerebral Ischemia (eTICI) grade ≥2c (11). Following the procedure, patients received antithrombotic bridging therapy with tirofiban, followed by dual antiplatelet therapy (aspirin 100 mg/day plus clopidogrel 75 mg/day) for a minimum of 3 months.

### 2.5 Outcome measures

The primary outcome was the 90-day mRS score distribution, with functional disability (mRS ≥ 3) as a dichotomized secondary endpoint; stroke or death within 30 days was a safety outcome. The secondary outcomes in the EVT subgroup included intracranial hemorrhage within 30 days (identified on routine CT at 24 h after the procedure), new cerebral infarction (confirmed by diffusion-weighted imaging [DWI] hyperintensity), and in-stent restenosis (stenosis > 50% within the stent or within 5 mm of the stent edges on 30-day follow-up imaging). Early neurological deterioration (secondary outcome) was defined as an increase of ≥4 points in the NIHSS during hospitalization, occurring after completion of the baseline assessment.

### 2.6 Statistical analysis

Analyses were performed using SPSS 27.0; proportional-odds analyses and between-score comparisons were performed using R 4.6.1 (MASS 7.3–65, rms 8.1–1, nnet 7.3–20, and pROC 1.19.0.1). Continuous variables were expressed as mean ± SD or median (interquartile range [IQR]) and were compared using Student’s *t*-test or the Mann–Whitney U test, as appropriate. Categorical variables were summarized as counts and percentages and were compared using the chi-squared test or Fisher’s exact test. The 90-day mRS score distribution was compared between the risk groups using the Mann–Whitney U test in the overall cohort. Its adjusted association with the continuous score was examined using a proportional-odds model including admission NIHSS; the proportional-odds assumption was assessed using a likelihood-ratio comparison with a multinomial model, and common ORs with 95% CIs were reported.

This was an exploratory analysis of the score’s discriminative ability; no predictive model was developed or calibrated, and the score constituted a prespecified, effect-size–anchored weighted system rather than a coefficient-derived model. Its independent association with 90-day disability (mRS ≥ 3) was tested using univariable and multivariable logistic regression analyses, reported as ORs with 95% CIs. Given only 22 disability events, covariates were limited based on events-per-variable considerations, with admission NIHSS prespecified as the principal confounder; the score was entered as a continuous variable and, in a sensitivity analysis, dichotomized at ≥8 points.

Discrimination was evaluated using an ROC analysis. The optimal cutoff was defined by the maximum Youden index. The score was applied without coefficient, item-weight, or threshold refitting in this cohort. Incremental discrimination over the original ABCD^3^-I score was evaluated using ΔAUC with its 95% CI and the DeLong test. A prespecified sensitivity analysis excluded the stenosis component, which was constant at 2 points in this ≥70% cohort.

All tests were two-sided, with a *p*-value of < 0.05 considered significant. Because this study was hypothesis-generating, no adjustment for multiplicity was applied, and all *p*-values should be interpreted as exploratory.

## 3 Results

### 3.1 Baseline characteristics

A total of 133 patients were included in the final analysis. Table 2 summarizes the baseline demographic and clinical characteristics of patients in the high-risk and non–high-risk groups. There were no significant differences between the two groups with respect to age, hyperlipidemia, hyperhomocysteinemia, coronary artery disease, atrial fibrillation, or hypertension (all *p* > 0.05). Atrial fibrillation was analyzed using Fisher’s exact test because the minimum expected cell count was less than 5 (minimum expected count: 3.50). Compared with the non-high-risk group, the high-risk group had a significantly higher NIHSS score at admission (median 2.0 [IQR 1.0–4.0] vs. 0.0 [IQR 0.0–1.0]; z = −4.951, *p* < 0.001) and a higher proportion of male patients (81.4% vs. 64.5%; χ^2^ = 3.867, *p* = 0.049), diabetes mellitus (43.1% vs. 19.4%; χ^2^ = 5.732, *p* = 0.017), current alcohol use (24.5% vs. 6.5%; χ^2^ = 4.792, *p* = 0.029), and current smoking (46.1% vs. 19.4%; χ^2^ = 7.083, *p* = 0.008).

**Table 2:** Comparison of baseline characteristics between high-risk and non-high-risk groups.

Variable	High-risk (*n* = 102)	Non-high-risk (*n* = 31)	Statistic	*p*-value
Age, years (mean ± SD)	65.26 ± 10.14	61.94 ± 11.69	*t* = 1.543	0.125
Male sex, *n* (%)	83 (81.4)	20 (64.5)	χ^2^ = 3.867	0.049
Diabetes mellitus, *n* (%)	44 (43.1)	6 (19.4)	χ^2^ = 5.732	0.017
Hyperlipidemia, *n* (%)	62 (60.8)	15 (48.4)	χ^2^ = 1.499	0.221
Hyperhomocysteinemia, *n* (%)	50 (49.0)	10 (32.3)	χ^2^ = 2.698	0.101
Current alcohol use, *n* (%)	25 (24.5)	2 (6.5)	χ^2^ = 4.792	0.029
Current smoking, *n* (%)	47 (46.1)	6 (19.4)	χ^2^ = 7.083	0.008
Coronary artery disease, *n* (%)	33 (32.4)	9 (29.0)	χ^2^ = 0.121	0.728
Atrial fibrillation, *n* (%)	12 (11.8)	3 (9.7)	-	>0.999
Admission NIHSS, median (IQR)	2.0 (1.0, 4.0)	0.0 (0.0, 1.0)	*z* = −4.951	<0.001
Hypertension, *n* (%)	78 (76.5)	20 (64.5)	χ^2^ = 1.752	0.186

NIHSS, National Institutes of Health Stroke Scale; IQR, interquartile range; SD, standard deviation. For atrial fibrillation, “-” indicates that Fisher’s exact test was used (minimum expected cell count = 3.50).

### 3.2 Primary outcome: 90-day mRS score distribution in the whole cohort

In the overall cohort, the unadjusted 90-day mRS score distribution was worse in the high-risk group (median 2.0 [IQR 1.0–2.0] vs. 1.0 [IQR 0.0–1.5]; mean rank 71.25 vs. 53.02; z = −2.405, *p* = 0.016). In the proportional-odds model, the score was associated with a worse mRS score before adjustment (common OR 1.25 per point, 95% CI 1.08–1.46; *p* = 0.004) but not after adjustment for admission NIHSS (adjusted common OR 1.05, 95% CI 0.88–1.26; *p* = 0.573), whereas NIHSS remained significant (adjusted common OR 1.24 per point, 95% CI 1.11–1.40; *p* < 0.001); the proportional-odds assumption was not rejected (*p* = 0.070). The category-specific distributions are shown in Table 3.

**Table 3:** Distribution of 90-day modified Rankin Scale (mRS) scores by risk group.

90-day mRS score	High-risk (*n* = 102)	Non-high-risk (*n* = 31)
0	15	9
1	35	14
2	33	5
3	10	0
4	6	2
5	3	1
6	0	0

The 90-day disability rate was 18.6% (19/102) in the high-risk group (score ≥8) and 9.7% (3/31) in the non–high-risk group (score <8); this difference in the dichotomized disability rate (mRS ≥ 3) did not reach statistical significance (Fisher’s exact test, *p* = 0.285), in contrast to the significant difference in the full ordinal mRS distribution described above (Table 3).

### 3.3 Association between the score and early neurological deterioration

**Figure 1:**
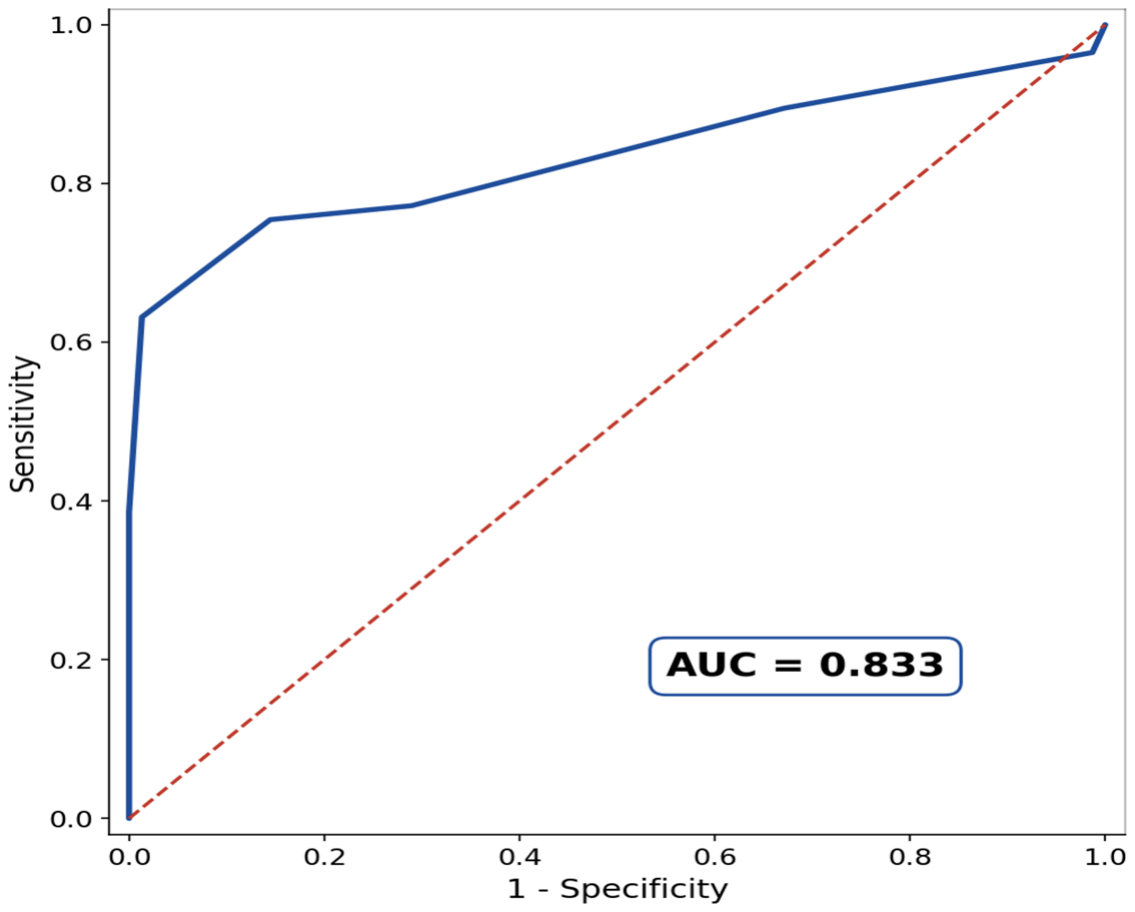
Receiver operating characteristic curve of the ABCD^3^-I-informed score for predicting early neurological deterioration (END; *n* = 133, 57 events). The solid line represents the ROC curve, and the dashed line indicates the reference line of no discrimination. AUC 0.833 (95% CI 0.760–0.906; *p* < 0.001). AUC, area under the curve.

Early neurological deterioration (END), defined as an increase of ≥4 points in the NIHSS during hospitalization, occurred in 57 of 133 patients (42.9%). The score was significantly associated with END, with an AUC of 0.833 (95% CI 0.760–0.906; *p* < 0.001). END was significantly more frequent in the high-risk group than in the non-high-risk group (51/102 [50.0%] vs. 6/31 [19.4%]; χ^2^ = 7.909, *p* = 0.005) (Table 4; Figure 1).

**Table 4:** Association between the score and early neurological deterioration (END).

Analysis	Result
Overall END incidence, *n* (%)	57/133 (42.9)
Score predicting END (ROC analysis)	
AUC (95% CI)	0.833 (0.760–0.906)
*p*-value	<0.001
END by risk group, *n* (%)	
High-risk group (score ≥8)	51/102 (50.0)
Non-high-risk group (score <8)	6/31 (19.4)
χ^2^	7.909
*p*-value	0.005

END, early neurological deterioration (defined as an increase of ≥4 points in the NIHSS during hospitalization); AUC, area under the curve; ROC, receiver operating characteristic; CI, confidence interval; NIHSS, National Institutes of Health Stroke Scale.

### 3.4 Short-term outcomes in the IMT subgroup

In the IMT subgroup, dichotomized disability rates did not differ significantly between high-risk and non-high-risk patients (18.5% vs. 10.0%; *p* = 0.494). Two 30-day strokes occurred in high-risk patients, and none occurred in non-high-risk patients; no deaths occurred (Table 5).

**Table 5:** Short-term outcomes in the intensive medical therapy subgroup.

Outcome	High-risk (*n* = 54)	Non-high-risk (*n* = 20)	Statistic	*p*-value
Disability (mRS ≥ 3), *n* (%)	10 (18.5)	2 (10.0)	-	0.494
Any stroke within 30 days, *n* (%)	2 (3.7)	0 (0.0)	-	>0.999
Death within 30 days, *n* (%)	0 (0.0)	0 (0.0)	-	-

mRS, modified Rankin Scale. Fisher’s exact test was used for all dichotomized outcomes.

### 3.5 Short-term outcomes in the EVT subgroup

In the EVT subgroup, disability rates did not differ significantly between high-risk and non-high-risk patients (18.8% vs. 9.1%; *p* = 0.670). Thirty-day stroke occurred in five high-risk patients and one non-high-risk patient, with no deaths; additional adverse events are summarized in Tables 6, 7.

**Table 6:** Short-term outcomes in the endovascular treatment subgroup.

Outcome	High-risk (*n* = 48)	Non-high-risk (*n* = 11)	Statistic	*p*-value
Disability (mRS ≥ 3), *n* (%)	9 (18.8)	1 (9.1)	-	0.670
Any stroke within 30 days, *n* (%)	5 (10.4)	1 (9.1)	-	>0.999
Death within 30 days, *n* (%)	0 (0.0)	0 (0.0)	-	-
Intracranial hemorrhage within 30 days, *n* (%)	2 (4.2)	1 (9.1)	-	0.468
New cerebral infarction within 30 days, *n* (%)	3 (6.3)	0 (0.0)	-	>0.999
In-stent restenosis within 30 days, *n* (%)	2 (4.2)	0 (0.0)	-	>0.999

mRS, modified Rankin Scale. Fisher’s exact test was used for all dichotomized outcomes.

**Table 7:** Summary of post-treatment adverse cerebrovascular events in the EVT subgroup.

No.	Group	Event type	Severity	Timing	Management	Outcome
1	High-risk	Intracranial hemorrhage	Non-fatal	In-hospital post-procedure	Conservative management	Clinically stable
2	High-risk	Intracranial hemorrhage	Non-fatal	In-hospital post-procedure	Conservative management	Clinically stable
3	Non-high-risk	Intracranial hemorrhage	Non-fatal	In-hospital post-procedure	Conservative management	Clinically stable
4	High-risk	Cerebral infarction	Non-fatal	In-hospital post-procedure	Intensified antiplatelet + neuroprotective therapy	Symptom improvement
5	High-risk	Cerebral infarction	Non-fatal	In-hospital post-procedure	Intensified antiplatelet + neuroprotective therapy	Symptom improvement
6	High-risk	In-stent restenosis with symptomatic cerebral infarction	Non-fatal	Within 30 days (follow-up imaging)	Intensified antiplatelet + neuroprotective therapy	Symptom improvement
7	High-risk	In-stent restenosis	Non-fatal	Within 30 days (follow-up imaging)	Continued surveillance	No deterioration

### 3.6 ROC analysis and cutoff performance

The score showed modest discrimination for 90-day disability (AUC 0.662, 95% CI 0.540–0.783; *p* = 0.017). At the prespecified ≥8-point threshold, sensitivity was 86.4%, specificity was 25.2%, positive predictive value (PPV) was 18.6%, and negative predictive value (NPV) was 90.3% (95% CI 75.1–96.7%, Wilson method). Operating characteristics for the prespecified ≥8-point and *post-hoc* Youden-optimal ≥9-point thresholds are presented in Table 8.

**Table 8:** Classification performance of the score at two cutoff thresholds for predicting 90-day disability (mRS ≥ 3).

Cutoff	Sensitivity	Specificity	PPV	NPV	Youden index
≥8 points (prespecified high-risk threshold)	86.4%	25.2%	18.6%	90.3% (75.1–96.7)	0.116
≥9 points (Youden-optimal)	77.3%	56.8%	26.2%	92.6% (83.9–96.8)	0.340

PPV, positive predictive value; NPV, negative predictive value. NPV 95% CI calculated using the Wilson score method. Base rate (non-disability rate): 83.5% (111/133).

Excluding the constant stenosis component did not change the AUC (0.662) or risk-group assignment after the corresponding threshold was shifted from ≥8 to ≥6 points.

**Figure 2:**
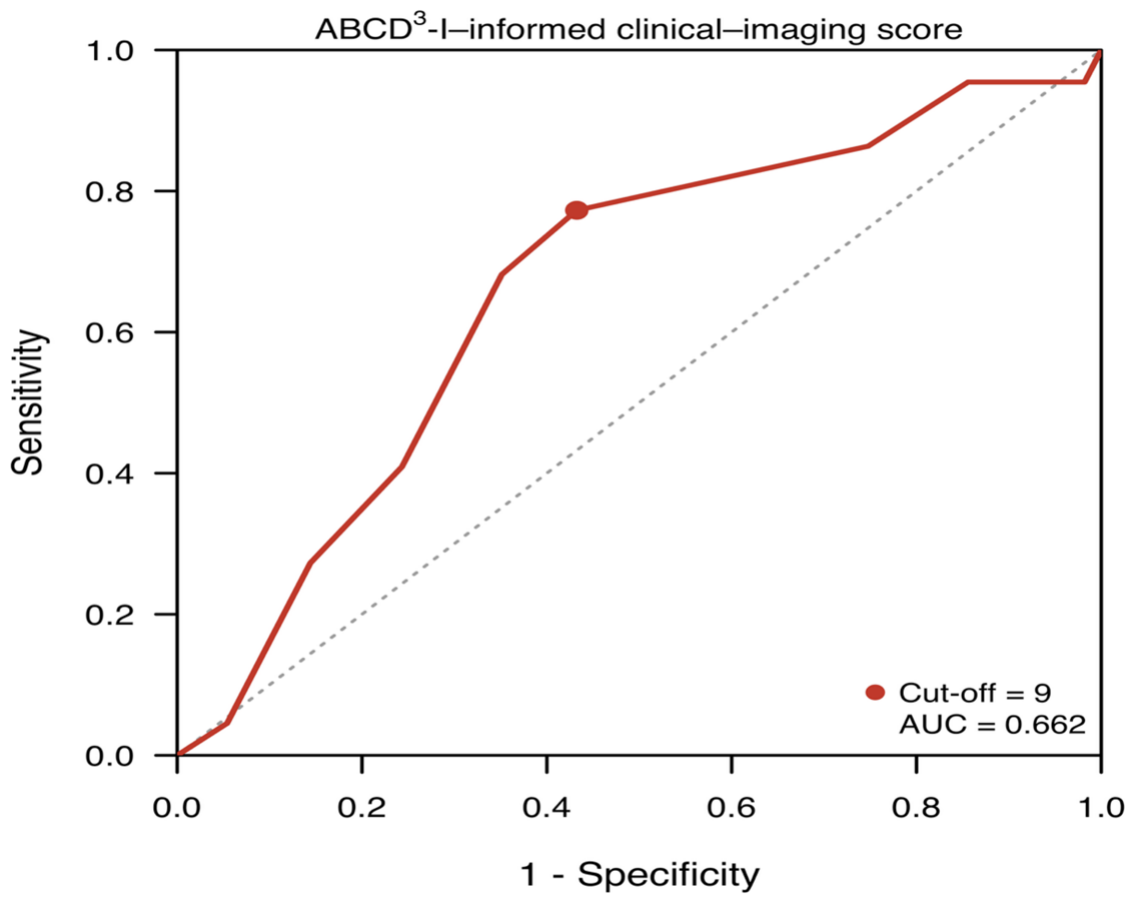
ROC curve of the ABCD^3^-I-informed score for predicting 90-day disability (mRS ≥ 3). The red solid line represents the ROC curve, and the gray dashed line indicates the reference line of no discrimination. The filled circle marks the optimal cutoff point (score = 9) as determined by the Youden index, yielding a sensitivity of 77.3% and a specificity of 56.8%. AUC, area under the curve.

The ≥8-point threshold was prespecified before the outcome analysis, whereas the Youden-optimal ≥9-point threshold was identified *post hoc* and was reported as a sensitivity analysis (Table 8). The optimal operating threshold should be re-evaluated in prospective cohorts (Figure 2).

### 3.7 Multivariable association between the score and 90-day disability

In the univariable analysis, both the score (crude OR 1.30 per point, 95% CI 1.04–1.63, *p* = 0.023) and admission NIHSS (crude OR 1.25, 95% CI 1.10–1.42, *p* < 0.001) were significantly associated with 90-day disability, whereas diabetes, smoking, alcohol use, and sex were not significantly associated (all *p* > 0.05). In the multivariable model including the score (continuous) and admission NIHSS, admission NIHSS remained independently associated with 90-day disability (adjusted OR 1.22, 95% CI 1.06–1.40, *p* = 0.005), whereas the association of the score was attenuated and no longer statistically significant (adjusted OR 1.11 per point, 95% CI 0.86–1.44, *p* = 0.413). A sensitivity analysis using the ≥8-point dichotomy yielded consistent results (score ≥8: adjusted OR 1.24, 95% CI 0.31–4.92, *p* = 0.759; admission NIHSS: adjusted OR 1.25, 95% CI 1.10–1.42, *p* < 0.001) (Table 9).

**Table 9:** Univariable and multivariable logistic regression analyses for 90-day disability (mRS ≥ 3).

Variable	Crude OR (95% CI)	*p*	Adjusted OR (95% CI)	*p*
ABCD^3^-I-informed score (per point)	1.30 (1.04–1.63)	0.023	1.11 (0.86–1.44)	0.413
Admission NIHSS (per point)	1.25 (1.10–1.42)	<0.001	1.22 (1.06–1.40)	0.005
Diabetes	1.18 (0.47–3.01)	0.726	—	—
Smoking	1.32 (0.52–3.31)	0.557	—	—
Alcohol use	0.85 (0.26–2.76)	0.787	—	—
Sex (male)	0.43 (0.16–1.16)	0.096	—	—

The multivariable model included the score and admission NIHSS (two covariates; events-per-variable ≈ 11). OR, odds ratio; CI, confidence interval; mRS, modified Rankin Scale. Dashes indicate variables not entered into the multivariable model.

### 3.8 Incremental discriminative value

**Figure 3:**
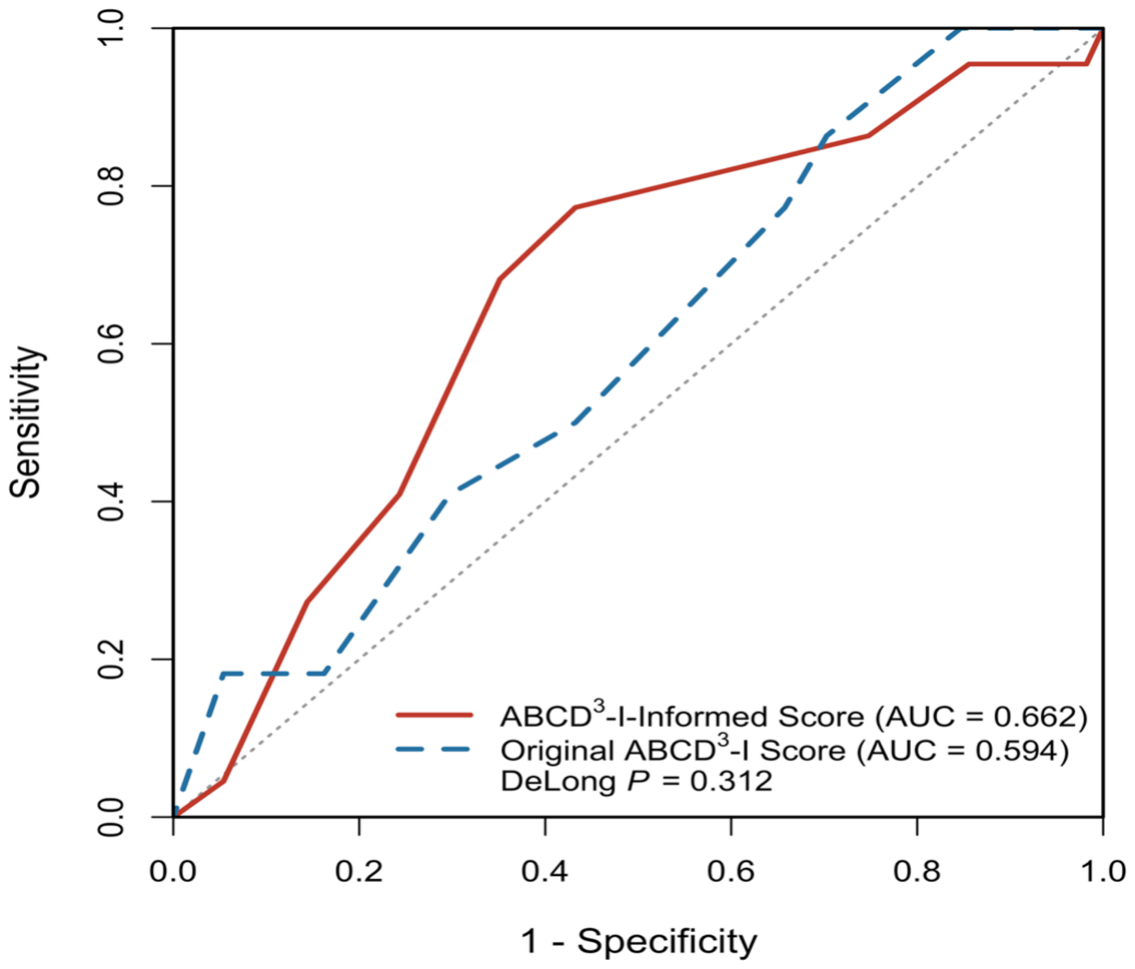
ROC curves of the ABCD^3^-I-informed score versus the original ABCD^3^-I score for predicting 90-day disability (*mRS* ≥ 3). The ABCD^3^-I-informed score (AUC 0.662) showed numerically higher discrimination than the original ABCD^3^-I score (AUC 0.594); the difference was not statistically significant (DeLong *p* = 0.312).

Compared with the original ABCD^3^-I score (AUC 0.594), the ABCD^3^-I-informed score showed a numerically higher AUC (0.662), with a ΔAUC of 0.068 (95% CI − 0.063 to 0.196; DeLong *p* = 0.312). The ΔAUC was not statistically significant, indicating that an incremental improvement in discrimination was not confirmed in this limited sample (Figure 3).

### 3.9 Descriptive comparison with the SAMMPRIS trial

As a purely descriptive benchmark, the 30-day stroke or death rates in our IMT (2.7%) and EVT (10.2%) cohorts were numerically lower than those in the medical (5.8%) and stenting (14.7%) arms of the Stenting and Aggressive Medical Management for Preventing Recurrent Stroke in Intracranial Stenosis (SAMMPRIS) trial, with no 30-day mortality. These figures are provided for context only. Because SAMMPRIS differed substantially from this study in design, eligibility criteria, patient characteristics, treatment allocation, and follow-up methodology, no inference regarding comparative efficacy or superiority can be drawn. Detailed figures are provided in Table 10.

**Table 10:** Descriptive comparison of 30-day safety outcomes between the present cohort and the SAMMPRIS trial.

Outcome	Present IMT (*n* = 74)	SAMMPRIS medical (*n* = 227)	Present EVT (*n* = 59)	SAMMPRIS PTAS (*n* = 224)
Any stroke within 30 days, *n* (%)	2 (2.7)	12 (5.3)	6 (10.2)	33 (14.7)
Stroke or death within 30 days, *n* (%)	2 (2.7)	13 (5.8)	6 (10.2)	33 (14.7)
Death within 30 days, *n* (%)	0 (0.0)	1 (0.4)	0 (0.0)	5 (2.2)
Intracranial hemorrhage within 30 days, *n* (%)	0 (0.0)	0 (0.0)	3 (5.1)	10 (4.5)
Ischemic stroke in territory of qualifying artery within 30 days, *n* (%)	2 (2.7)	10 (4.4)	3 (5.1)	23 (10.3)

SAMMPRIS data from reference (14).

## 4 Discussion

In this exploratory cohort, the score was associated with worse unadjusted ordinal 90-day mRS but not after adjustment for admission NIHSS. It showed strong discrimination for END, consistent with its END-oriented construction, but only modest discrimination for 90-day disability, and no significant incremental gain over the original ABCD^3^-I score.

Watershed (border-zone) infarction in intracranial atherosclerotic disease (ICAD) is biologically plausible as a marker of hemodynamic vulnerability: severe responsible-vessel stenosis reduces distal perfusion, and inadequate collateral compensation preferentially affects border-zone territories (8, 9, 12). This supports the inclusion of DWI-defined watershed infarction and stenosis severity. However, the stenosis component was constant in this ≥70% cohort and could not contribute to within-cohort discrimination. Moreover, the re-specified imaging variables were selected from prior associations with END, while progressive stroke is conceptually related, although temporally distinct, to subsequent END; the stronger END performance should therefore be interpreted as construct consistency rather than independent validation.

The discordance between ordinal and dichotomized findings may reflect information loss and limited power. Discrimination for 90-day disability was modest (AUC 0.662, 95% CI 0.540–0.783); because the lower confidence bound approaches 0.5, clinically non-informative performance is possible. The score should not be used as a stand-alone predictive instrument. The prespecified ≥8-point threshold prioritized sensitivity (86.4%) and yielded an NPV of 90.3%, whereas the *post-hoc* Youden-optimal ≥9-point threshold had higher specificity (56.8%) and NPV (92.6%). These findings suggest greater potential for identifying a non-high-risk subgroup than for confirming high risk; however, given the wide confidence intervals and only 22 disability events, both thresholds remain exploratory and require prospective validation.

After adjustment for admission NIHSS, neither the ordinal nor the dichotomized association remained significant, suggesting that much of the score’s prognostic information overlaps with baseline stroke severity. The score should therefore complement, not replace, the NIHSS and should not determine treatment modality.

The non-significant ΔAUC indicates that superiority over the original ABCD^3^-I score was not demonstrated rather than proving equivalence. Multicenter validation, calibration assessment, and decision curve analysis remain necessary.

Unlike Angio-FF (13), which requires DSA and distinguishes symptomatic from asymptomatic stenosis, the present score uses information routinely available at admission and addresses short-term risk stratification. The two approaches should therefore be regarded as complementary rather than interchangeable.

This study has several limitations. First, the retrospective single-center design and exclusion of six patients may have introduced selection or attrition bias. Treatment was non-randomized, the non-high-risk EVT subgroup was small (*n* = 11), and high-risk patients were disproportionately allocated to EVT, creating potential confounding by indication. Treatment-stratified findings are therefore descriptive and cannot support comparative effectiveness or IMT/EVT selection. Second, statistical precision was limited by the sample size (*n* = 133), 22 disability events, and only 31 non-high-risk patients. At the ≥8-point threshold, the NPV of 90.3% was calculated from 31 non-high-risk patients, three of whom experienced disability (95% CI 75.1–96.7%); 76.7% of patients were classified as high risk, and specificity was 25.2%. Thus, high-risk classification is not a strong positive predictor and should at most prompt closer monitoring. Third, restriction to severe anterior circulation stenosis (70–99%) and a single high-volume center limits generalizability to moderate or posterior circulation disease and other settings; the 90-day follow-up also precludes assessment of long-term. Outcomes Finally, the score used pragmatic rather than coefficient-derived weights; coefficient-based reweighting, external calibration, and clinical-utility assessment should be evaluated in larger prospective multicenter cohorts.

## 5 Conclusion

In this independent exploratory cohort, integrating pre-baseline clinical instability with routinely available DWI and stenosis features provided a mechanistically grounded framework for characterizing early risk in severe anterior circulation ICAS. The ABCD^3^-I-informed score showed strong END discrimination, consistent with its END-oriented construction, and its high negative predictive value suggests potential for identifying a non-high-risk subgroup. However, its modest, NIHSS-dependent 90-day performance and non-significant incremental gain over the original ABCD^3^-I score preclude its use as a stand-alone prognostic or treatment-selection tool. The principal contribution of this study is therefore to define a clinically accessible candidate framework and its current performance limits, providing a basis for model refinement and prospective multicenter evaluation across a broader stenosis spectrum.

## Data Availability

The raw data supporting the conclusions of this article will be made available by the authors, without undue reservation.
